# A case of chronic eosinophilic pneumonia associated with rheumatoid arthritis in glucocorticoid-free remission with JAK inhibitor: A case report

**DOI:** 10.1097/MD.0000000000033396

**Published:** 2023-03-31

**Authors:** Takashi Yamane, Akira Hashiramoto

**Affiliations:** a Department of Rheumatology, Kakogawa Central City Hospital, Kakogawa, Japan; b Department of Biophysics, Kobe University Graduate School of Health Sciences, Kobe, Japan.

**Keywords:** chronic eosinophilic pneumonia, glucocorticoids, Janus kinase inhibitors, rheumatoid arthritis

## Abstract

Chronic eosinophilic pneumonia (CEP) presents eosinophil infiltrations in the lung due to allergic reactions. Most CEP patients continue to take glucocorticoids, and their prolonged use induces various side effects. In this case report, based on the efficacy of baricitinib in patients with rheumatoid arthritis (RA) and CEP, we aimed to show that the administration of Janus kinase (JAK) inhibitors, when RA is complicated by an allergic disease, can stabilize the disease state and help avoid the adverse effects of long-term systemic glucocorticoid administration.

**Patients concerns:**

A 56-year-old woman developed RA at the age of 19 years. Treatment of the arthritis was initiated, but the joint destruction had progressed. At the age of 42, she developed eosinophilic pneumonia, which was relieved by glucocorticoid therapy. Since then, maintenance therapy has been continued with the diagnosis of CEP. She was treated with concomitant tacrolimus for persistent arthritis, and the prednisolone (PSL) dose was reduced to 3 mg/day after 10 years. However, around this time, an increase in peripheral blood eosinophil counts and respiratory symptoms was observed.

**Diagnosis:**

The peripheral blood eosinophil count was 4000/µL and computed tomography revealed multiple ground-glass opacities in the peripheral lung fields. As interstitial pneumonia due to infection or other causes was ruled out, CEP relapse was diagnosed.

**Interventions:**

Pneumonia rapidly recovered when the PSL dose was increased to 15 mg/day, and asymptomatic eosinophilic infiltrates reappeared in the lung field along with a relapse of arthritis when the PSL dose was reduced to 5 mg/day. Concomitant use of methotrexate and baricitinib has been introduced to suppress allergic reactions to pneumonia.

**Outcomes:**

After starting combination therapy with baricitinib and methotrexate, both arthritis and eosinophilia improved, and glucocorticoid-free remission was achieved.

**Lessons:**

Recently, inhibition of IL-5 signaling via JAK2 has been reported to be effective in bronchial asthma and atopic dermatitis. Although complications of RA and CEP are not common, the actions of baricitinib are useful not only in arthritis but also in allergic diseases. The efficacy of some JAK inhibitors should be actively tested in patients with RA and these complications.

## 1. Introduction

Chronic eosinophilic pneumonia (CEP) is a disease of unknown etiology that was first described in 1969 and is characterized by eosinophil infiltrations in the lungs. The disease commonly affects middle-aged women and presents with clinical symptoms, such as cough, fever, and dyspnea. Chest computed tomography is considered highly useful in the diagnosis of CEP; however, the exclusion of infection is essential for a definitive diagnosis. Approximately half of the patients with CEP have atopic diseases such as bronchial asthma, and allergic reactions are thought to be involved in the disease development.^[1]^ Similar to CEP, acute eosinophilic pneumonia is also characterized by eosinophil infiltration into the lungs, while acute eosinophilic pneumonia is unique in that it is caused by smoking and can often achieve remission without treatment.^[2]^ Although glucocorticoid therapy has shown marked efficacy in CEP, there is insufficient evidence regarding the optimal duration of treatment. Therefore, approximately half of the patients relapse after tapering or discontinuation of treatment and are forced to continue maintenance therapy. Some cases lead to pulmonary fibrosis; however, most cases, including recurrent cases, respond well to glucocorticoid therapy.^[3]^ Needless to say, adverse events associated with long-term glucocorticoid therapy are detrimental to the patient’s quality of life and should be discontinued or minimized as much as possible.^[4,5]^

Rheumatoid arthritis (RA) is a systemic autoimmune disease characterized by inflammatory synovitis and progressive joint destruction. Recently, the activated Janus kinase (JAK)/signal transducer and activator of the transcription pathway has been shown to play an important role in intracellular signaling from the cell surface, and it can dramatically reduce the disease activity of RA by inhibiting multiple cytokines signaling. The JAK inhibitor baricitinib exerts its clinical efficacy in the treatment of RA by inhibiting JAK1/2. Compared to conventional methotrexate (MTX), baricitinib can slow the progression of structural damage even in patients with moderate to high disease activity.^[6]^ Recently, inhibition of IL-5 signaling via JAK2 has been reported to be effective in bronchial asthma and atopic dermatitis by suppressing eosinophil differentiation and proliferation.^[7,8]^ Atopic diseases, including asthma and dermatitis, have been reported to be associated with autoimmune diseases such as RA.^[9,10]^

Here, we report a case of steroid-dependent CEP secondary to RA in which baricitinib was effective in both treatments and achieved glucocorticoid-free remission.

## 2. Case presentation

**Figure 1.**
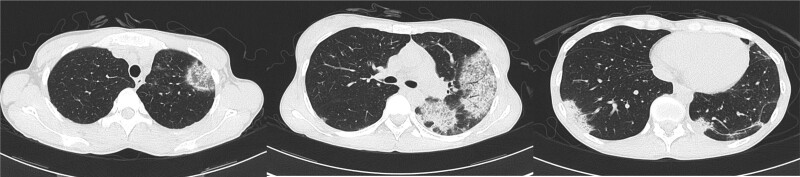
Chest computed tomography showing multiple bilateral peripheral patchy ground-glass opacities.

**Figure 2.**
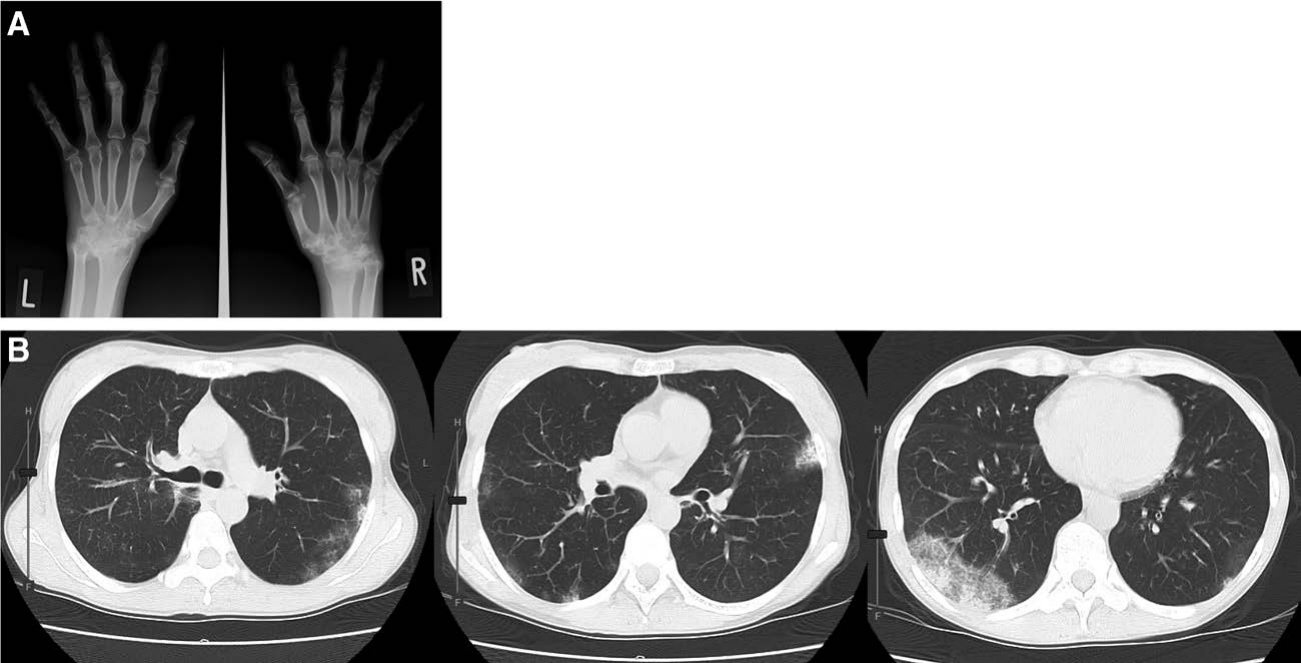
(A) X-ray of both hands showing loss of intercarpal and carpometacarpal joint spaces, ulna deviation of MCP joints, and deformity of the PIP joint of the left middle finger. (B) Chest computed tomography showing bilateral ground-glass opacities, similar to initial findings. MCP = metacarpophalangeal, PIP = proximal interphalangeal.

**Figure 3.**
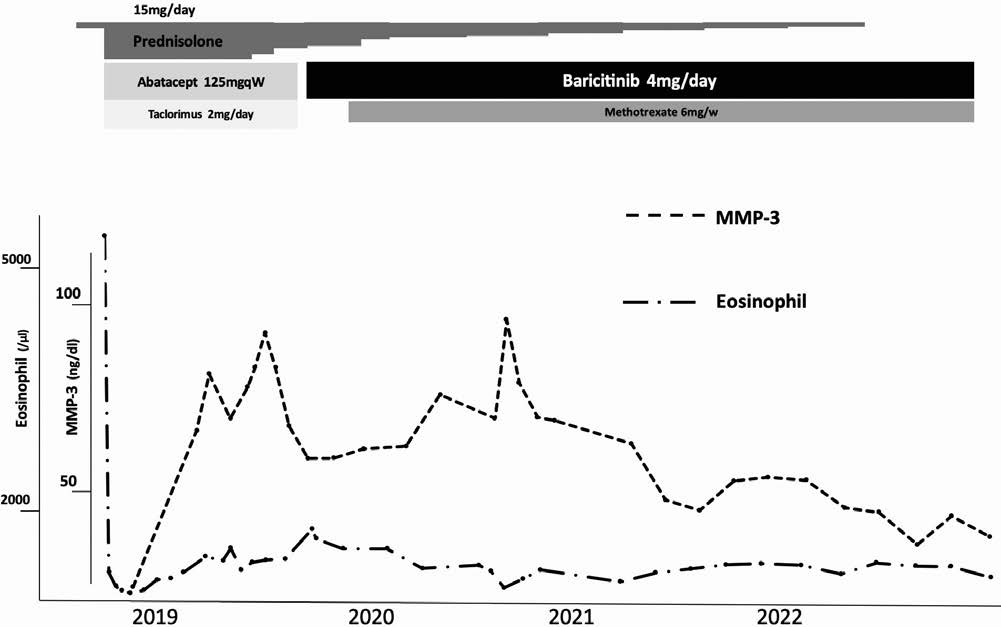
Clinical course.

A 56-year-old Japanese woman developed seropositive RA at 19 years of age. Treatment of arthritis initially started with an intramuscular injection of gold while joint destruction, especially in both hands, progressed. In 2008, at the age of 42 years, she developed eosinophilic pneumonia without any triggers (Fig. 1), which was relieved by intravenous methylprednisolone pulse therapy. Since then, maintenance therapy with prednisolone (PSL) has been continued with the diagnosis of CEP. Although abatacept (ABT) was introduced for the treatment of RA, she underwent bilateral total knee arthroplasty owing to progressive joint deformities. One year after surgery she developed septic arthritis of the right knee and ABT was discontinued to replace glucocorticoid monotherapy. Thereafter, for persistent arthritis, she was treated with concomitant tacrolimus, and PSL was reduced from 3.5 to 3 mg/day in 2018. However, around this time, an increase in peripheral blood eosinophil counts and respiratory symptoms appeared, and the patient was transferred to our department with a suspected CEP relapse. Radiography of the hands showed typical findings of bilaterally symmetric, destructive, and erosive RA, especially in the wrist joints (Fig. 2A). The peripheral blood eosinophil count was 4000/µL, and the serum KL-6 level was as high as 637 IU/ml. Computed tomography images revealed multiple ground-glass opacities in the peripheral lung fields (Fig. 2B). As interstitial pneumonia due to infection or other causes was ruled out, CEP relapse was diagnosed. Pneumonia rapidly recovered when the PSL dose was initially increased to 15 mg/day. However, in 2019, asymptomatic eosinophilic infiltrates reappeared in the lung field along with a relapse of arthritis when the PSL dose was reduced to 5 mg/day. The eosinophilia persisted though ABT was restarted, and concomitant use of MTX and baricitinib 4 mg/day was started because of the expected effect of the JAK inhibitor on hypereosinophilia. After starting the combination therapy with baricitinib and MTX, both arthritis and eosinophilia improved, and glucocorticoid-free remission was finally achieved in 2022 (Fig. 3).

## 3. Discussion

Here, we reported a case of CEP secondary to RA that was successfully treated with baricitinib. Regardless of the underlying disease, long-term administration of systemic glucocorticoids should be avoided, considering drug-related adverse events. In the case of bronchial asthma, it has been reported that the risk of any complications due to glucocorticoid is 2.5 times higher even at a PSL of 5 mg/day or less.^[11]^ Even a small amount of glucocorticoids increases the risk of fracture and cardiovascular risk.^[4,5]^

An anti-IL-5 agent has been reported to cease systemic glucocorticoid administration for CEP. However, it is not only ineffective for RA but also an anti-IL-5 agent that sometimes develops arthritis by reducing the concomitant glucocorticoid dose.^[12]^ Similarly, anti-IgE antibodies can also reduce glucocorticoid doses, but their effects are limited to cases with high IgE levels.^[13]^

Baricitinib, originally developed for the treatment of RA, inhibits cytokine signaling, including IL-6, IL-12, IL-20, IL-22, IL-23, and IFN-γ, but also exerts regulatory effects on the Th2 cytokines such as IL-4, IL-5, and IL-13.^[7]^ Therefore, it appears to be a candidate drug with the potential to reduce glucocorticoid doses in both RA and CEP. Although complications of RA and CEP are uncommon,^[14]^ bronchial asthma and atopic dermatitis, similar allergic conditions have been suggested to be related to various autoimmune diseases^[9,15]^ and are also a risk factor for developing RA.^[10]^ Furthermore, bronchial asthma has been reported to be associated with anticyclic citrullinated peptide antibody production.^[16]^

## 4. Conclusion

The JAK1/2 inhibitor baricitinib is a good treatment option for patients with eosinophil-related diseases such as CEP, which can simultaneously suppress both allergic reactions and inflammation to break glucocorticoid dependence. Some types of JAK inhibitors, such as baricitinib, may be considered effective treatments for RA patients with asthma or atopic dermatitis.

## Acknowledgments

We thank the patient who agreed to participate in this study.

## Author contributions

**Conceptualization:** Takashi Yamane.

**Data curation:** Takashi Yamane.

**Investigation:** Takashi Yamane.

**Resources:** Takashi Yamane.

**Supervision:** Akira Hashiramoto.

**Visualization:** Takashi Yamane.

**Writing – original draft:** Takashi Yamane.

**Writing – review & editing:** Takashi Yamane, Akira Hashiramoto.
